# No Association of Phenotypic ABO Blood Group and Malaria during Pregnancy

**DOI:** 10.4269/ajtmh.2012.12-0129

**Published:** 2012-09-05

**Authors:** Machteld E. Boel, Marcus J. Rijken, Mupawjay Pimanpanarak, Naw Lily Keereecharoen, Stephane Proux, François Nosten, Rose McGready

**Affiliations:** Shoklo Malaria Research Unit, Mae Sot, Tak, Thailand; Academic Medical Center, Amsterdam, The Netherlands; Faculty of Tropical Medicine, Mahidol University, Thailand; Centre for Tropical Medicine, Nuffield Department of Clinical Medicine, University of Oxford, United Kingdom

## Abstract

In a few small studies an association between blood group O and placental malaria has been described. The relationship between blood group and malaria in pregnancy (*Plasmodium vivax* and *Plasmodium falciparum*) was analyzed in 1,468 women from three longitudinal cohort studies in which weekly malaria screening was done systematically during pregnancy. One-third of women (447 of 1,468) had at least one malaria infection in pregnancy. The ABO blood group phenotype was not associated with the species of infection, frequency of malaria attacks, symptoms of malaria, hematocrit, or parasitemia during pregnancy.

## Introduction

Only a few studies have addressed the association between the ABO blood group system and malaria during pregnancy. Four studies from Africa (Gabon,^1^ The Gambia,^2^ Malawi,^3^ and Sudan^4^) were recently reported in a single work (the total number of women studied was 1,516: 378, 198, 647, and 293 women respectively).^1^ In The Gambia and Malawi, blood group O conferred a higher risk of active placental infection in primipara, but a significantly lower risk in multipara. These findings were not confirmed by the study from Gabon, in which no statistically significant risk of placental parasitemia was observed in any blood group.^1^ In another study in Sudan no association was found between blood group and active placental infection, which was observed in 18 of 236 women studied.^5^ We were unable to find any published reports from Asia. We determined the relationship between blood group and malaria (*Plasmodium vivax* and *Plasmodium falciparum*) in pregnancy and pregnancy outcome analyzing longitudinal data from three cohort studies in which regular malaria screening was done systematically during pregnancy.

## Methods

On the western border of Thailand, the Shoklo Malaria Research Unit has been conducting weekly antenatal clinics (ANC) since 1986. It has six established clinics that provide antenatal care and three clinics with delivery services for migrant workers and refugee populations from Myanmar. All pregnant women are encouraged to attend ANC on a weekly basis for malaria screening and early diagnosis and treatment, because there is no drug available for intermittent preventive treatment in this area of multi-drug resistant malaria.^6^

Since the ANC was established three pregnant women cohort studies have been conducted, all with frequent and longitudinal follow-up for malaria screening and routine blood group testing. In all studies exclusion criteria were limited to women who refused to participate in the trial. Two studies commenced enrollment from the first trimester and one study before 24 weeks in the second trimester. The blood group was obtained routinely in the time frame between enrollment and completion of the study. Attached to the ANC clinics were labor and delivery rooms, where women could deliver with trained midwifes. Birth weight was measured on electronic Seca scales (accuracy 10 grams) for unit deliveries and with Salter hanging scales (accuracy 50 g) for home deliveries.

### Statistical analysis.

Data were analyzed using SPSS for Windows version 18.0 (SPSS, Inc., Chicago, IL). In the analysis, women were categorized as “free of malaria” if all the malaria smears done at antenatal visits were negative, including malaria smears of the mother peripheral and placental blood at delivery. Women with a positive malaria smear were categorized into three groups: “*P. vivax* (only) group” or “*P. falciparum* (only) group” or both *P. falciparum* and *P. vivax* detected during pregnancy. The analysis of malaria in primipara and multipara was separated for the different species as differences with gravida groups and *P. vivax* have previously been reported.^7^ Proportions were compared using the χ^2^ test and the Bonferroni correction was applied for multiple comparisons. Birth weight was only used for analysis if the infant was weighed within 1 day after birth.

### Laboratory.

Blood smears (thin and thick films) were prepared using the Giemsa coloration and were read for 200 fields before declared negative. Parasite count was given by 500 white blood cells or by 1,000 red blood cells. All stages of the parasites were recorded (asexual and gametocytes). The blood group was tested with the agglutination test using serum anti-A and anti-B test (DiaMed; DiaClon monoclonal IgM antibody cell line G 1/2 and A5).

### Ethical approval.

All studies were approved by the Ethical Committee of the Faculty of Tropical Medicine, Mahidol University Bangkok, Thailand and Oxford Tropical Medicine Ethical Committee, Oxford University, England.

## Results

Of the 1,570 women included in the three cohorts 1,468 (93.5%) had a blood group phenotype available: group O was the most prevalent and group AB the least prevalent (Table 1). The 6.5% (102 of 1.570) of women who did not have blood group tested had a lower median number of antenatal visits (12 [1–32] versus 22 [1–41], *P* < 0.001), and were less likely to have been followed up until delivery (58% [59 of 102] versus 4% [62 of 1,468], *P* < 0.001) compared with women who were tested. Out of 1,468 women 447 (30.4%) had at least one malaria episode during pregnancy, and there was no significant difference in the proportion of women with malaria in the different blood groups (Table 2). The number of women with uncomplicated *P. falciparum* hyperparasitemia (eight cases), or severe *P. falciparum* malaria (one case), were so low that statistical comparisons were not performed. Of the 54% (793 of 1,570) women who delivered in the Shoklo Malaria Research Unit (SMRU) clinic, 487 (61.4%) placenta samples were available. Only 5 placentas (1.0%) were malaria positive: for blood group O 0.5% (1 of 205), A 0.9% (1 of 110), B 2.2% (3 of 137), and AB none of 35. Of the women who had malaria during pregnancy the proportion infected with different species was not significantly different between blood groups and this non-significant association was maintained when comparing symptomatic malaria infection, the median number of infections of *P. falciparum* or *P. vivax*, the geometric mean maximum parasitemia of *P. falciparum* or *P. vivax*, or the mean hematocrit at the time of *P. falciparum* or *P. vivax* (Table 3).

The proportion of primipara and multipara women with *P. falciparum* or *P. vivax* was not significantly different between blood groups (Table 3). The analysis was repeated for blood group O versus non-O, and blood group A versus non-A, and no significant differences were observed. The birth weight for all blood groups compared with each other, and for blood group O versus non-O, were not significantly different (data not shown).

## Discussion

This is the first study to examine the relationship between ABO blood group phenotype in women prospectively and actively followed for malaria (*P. falciparum* and *P. vivax*) during pregnancy. There was no indication from this large cohort where nearly one-third of women were diagnosed with malaria during pregnancy that ABO blood group phenotype relates to 1) the species of infection, 2) frequency of malaria attacks, 3) symptoms of malaria, 4) hematocrit, and 5) parasitemia. This absence of association remained true within each gravida group.

The findings presented are in contrast to those from The Gambia and Malawi, where a relationship between blood group O and placental malaria was observed.^2^,^3^ However, comparison with these studies is not straight forward, as the placental malaria was the main comparator to the blood group, whereas the main comparator in this study was (peripheral) malaria during pregnancy. However, peripheral infection is clearly related with placental malaria^8^,^9^; without an initial peripheral blood infection placental malaria cannot occur. In the most recent *P. falciparum* randomized controlled treatment trial in this population, placental infection only occurred in women with peripherally detected malaria infection that occurred at the time of delivery.^6^ In the SMRU clinics where pregnant women attend the weekly screening with early detection and treatment of malaria, placental malaria has become a rare event. The same applies for severe malaria, which is only seen in women who were absent from the weekly screening, and as a result there were insufficient numbers of women with placental or severe malaria to examine. This study suggests that ABO blood group phenotype does not affect the probability of peripheral parasitemia.

In addition, in this setting where immunity is low, symptoms and parasitemia are correlated with disease severity and these factors were also not significantly associated with ABO blood group phenotype. There was no relationship between birth weight and ABO blood group. This is in contrast to studies from The Gambia and Malawi, where besides a relationship with placental malaria an increased birth weight and a higher ponderal index were found in neonates from women with blood group O.^2^,^3^

This cohort is likely to be less subject to bias than previous publications as pregnant women were followed longitudinally and actively; therefore, the diagnosis of malaria did not depend upon whether a placenta was available or not. Despite the large number of women some of the sub-group analysis, especially for blood group AB, had only a small number of women. A much larger cohort from this population would be required to definitively rule out differences with blood group AB. This study does not support an association between blood group and malaria during pregnancy.

## Figures and Tables

**Table 1:** ABO blood group phenotype distribution among 1,468 pregnant women from three cohorts

	Year	Purpose of study	Blood group phenotype n (%)				
			O	A	B	AB	total
First cohort*	1998–2000	*P. vivax* prevention RCT CQ versus Placebo	192 (38.5)	113 (22.6)	168 (33.7)	26 (5.2)	499 (100.0)
Second cohort	2007–2009	Post-partum malaria susceptibility	293 (38.9)	179 (23.7)	230 (30.5)	52 (6.9)	754 (100.0)
Third cohort	2009–2011	Impact of MIP on fetal growth	96 (44.7)	50 (23.3)	54 (25.1)	15 (7.0)	215 (100.0)
Total			581 (39.6)	342 (23.3)	452 (30.8)	93 (6.3)	1468 (100.0)

* One thousand women recruited but only the placebo group was included here.^10^

CQ = chloroquine, MIP = malaria in pregnancy, RCT = randomized controlled trial.

**Table 2:** Phenotypic blood group and malaria

	ABO blood group phenotype, n (%)				*P* value*
	O	A	B	AB	
Any malaria					
No Malaria	408 (70.2)	239 (69.9)	314 (69.5)	60 (64.5)	NS
Malaria	173 (29.8)	103 (30.1)	138 (30.5)	33 (35.5)	
Species of malaria					
*P. falciparum* only	31 (17.9)	20 (19.4)	25 (18.1)	3 (9.1)	NS
*P. vivax* only	99 (57.2)	55 (53.5)	81 (58.7)	20 (60.0)	
Both *P. falciparum* and *P. vivax*	43 (24.9)	28 (27.2)	32 (23.2)	10 (30.3)	
Symptomatic malaria (at least once)					
Asymptomatic	74 (43.0)	46 (44.7)	50 (36.2)	14 (42.4)	NS
Symptomatic	98 (57.0)	57 (55.3)	88 (63.8)	19 (57.6)	
Median number of infections/pregnancy					
*P. falciparum* episodes	1 [1–4]	1 [1–3]	1 [1–4]	1 [1–3]	NS
*P. vivax* episodes	2 [1–6]	2 [1–7]	2 [1–8]	1 [1–6]	NS
Geometric mean parasitemia/uL					
*P. falciparum*	3,759 [16–194,805]	1,952 [16–915,588]	5,373 [32–311,458]	2,349 [112–74611]	NS
*P. vivax*	314 [16–62,734]	239 [16–41,448]	374 16–41,448]	380 [16–49,739]	NS
Mean hematocrit, %					
*P. falciparum*	30 [17–39]	32 [24–44]	31 [23–44]	30 [21–46]	NS
*P. vivax*	32 [17–43]	31 [34–45]	31 [19–39]	32 [17–45]	NS

* Bonferroni corrected; NS = non-significant.

**Table 3:** ABO blood group phenotype, malaria and parity

		ABO blood group phenotype				*P* value*
		O	A	B	AB	
*P. falciparum*						
Multigravida	No malaria	325 (93.9)	188 (92.6)	236 (92.2)	47 (94.0)	NS
	*P. falciparum*	21 (6.1)	15 (7.4)	20 (7.8)	3 (6.0)	
Primigravida	No malaria	82 (89.1)	51 (91.1)	78 (94.0)	13 (100.0)	NS
	*P. falciparum*	10 (10.9)	5 (8.9)	5 (6.0)	0	
*P. vivax*						
Multigravida	No malaria	325 (81.7)	188 (81.4)	236 (78.1)	47 (78.3)	NS
	*P. vivax*	73 (18.3)	43 (18.6)	66 (21.9)	13 (21.7)	
Primigravida	No malaria	82 (75.9)	51 (81.0)	78 (83.9)	13 (65.0)	NS
	*P. vivax*	26 (24.1)	12 (19.0)	15 (16.1)	7 (35.0)	

* Bonferroni corrected; NS = non-significant.
